# A Simple Guideline to Assess the Characteristics of RNA-Seq Data

**DOI:** 10.1155/2018/2906292

**Published:** 2018-11-04

**Authors:** Keunhong Son, Sungryul Yu, Wonseok Shin, Kyudong Han, Keunsoo Kang

**Affiliations:** ^1^Department of Microbiology, College of Natural Sciences, Dankook University, Cheonan 31116, Republic of Korea; ^2^Department of Clinical Laboratory Science, Semyung University, Jecheon 27136, Republic of Korea; ^3^Department of Nanobiomedical Science & BK21 PLUS NBM Global Research Center for Regenerative Medicine, Dankook University, Cheonan 31116, Republic of Korea

## Abstract

Next-generation sequencing (NGS) techniques have been used to generate various molecular maps including genomes, epigenomes, and transcriptomes. Transcriptomes from a given cell population can be profiled via RNA-seq. However, there is no simple way to assess the characteristics of RNA-seq data systematically. In this study, we provide a simple method that can intuitively evaluate RNA-seq data using two different principal component analysis (PCA) plots. The gene expression PCA plot provides insights into the association between samples, while the transcript integrity number (TIN) score plot provides a quality map of given RNA-seq data. With this approach, we found that RNA-seq datasets deposited in public repositories often contain a few low-quality RNA-seq data that can lead to misinterpretations. The effect of sampling errors for differentially expressed gene (DEG) analysis was evaluated with ten RNA-seq data from invasive ductal carcinoma tissues and three RNA-seq data from adjacent normal tissues taken from a Korean breast cancer patient. The evaluation demonstrated that sampling errors, which select samples that do not represent a given population, can lead to different interpretations when conducting the DEG analysis. Therefore, the proposed approach can be used to avoid sampling errors prior to RNA-seq data analysis.

## 1. Introduction

Recent advances in DNA sequencing technology led by next-generation sequencing (NGS) have been generating various molecular maps, including genomes, transcriptomes, and epigenomes [1–3]. Among them, NGS-based transcriptomic data called RNA-seq (or expression profiling by high throughput sequencing) is one of the most abundant data types according to statistics from the gene expression omnibus database (https://www.ncbi.nlm.nih.gov/geo/summary/?type=series) [4]. Since all living cells contain RNAs as messengers to convey protein-coding information from DNA to proteins or as a functional player such as noncoding RNAs, RNA-seq has become a major research tool for profiling various RNAs in many research fields. Technically, RNA-seq is based on sequenced reads from an NGS instrument [5]. Although researchers routinely check the sequencing quality of RNA-seq data using bioinformatics tools such as FastQC (https://www.bioinformatics.babraham.ac.uk/projects/fastqc/), it does not reflect the true quality (e.g., RNA quality) of RNA-seq data. In addition, no more than two or three biological replicates are generally used as representative samples for a population of a given condition (e.g., a disease), due to the high cost of RNA-seq or difficulty obtaining samples. This means that individual RNA-seq data will greatly affect the outcome. Potential sampling errors can be avoided by carefully examining RNA-seq data prior to downstream analysis such as differentially expressed gene (DEG) analysis. Here, we proposed a simple approach that provides a systematic view of a given RNA-seq dataset using two different principal component analysis (PCA) plots. The gene expression PCA plot provides a map of the distances between samples from which the characteristics of RNA-seq data can be inferred. However, the transcript integrity number (TIN) score [6] PCA plot can infer the quality (not the sequencing quality) of RNA-seq data, which can effectively discriminate low-quality samples. We proved method's usefulness by evaluating the RNA-seq data of ten invasive ductal carcinoma and three adjacent normal tissues biopsied from a Korean patient with breast cancer. Our approach clearly distinguished RNA-seq data according to gene expression levels and RNA qualities. The evaluation regarding the effect of incorporating a low-quality sample or a sample from a spatially distinct region on identifying differentially expressed genes (DEGs) showed that selecting the correct RNA-seq data to represent a population for a given condition significantly affected the identification of DEGs.

## 2. Materials and Methods

### 2.1. RNA-Seq Data Analysis

We had previously conducted RNA-seq with one tissue of invasive ductal carcinoma (luminal B subtype) from breast tissue and a corresponding adjacent normal tissue biopsied from a Korean breast cancer patient with informed consent. The data has been deposited under the gene expression omnibus (GEO) accession number GSE110114. We used this RNA-seq dataset to evaluate the impact of RNA quality and sample variations to identify differentially expressed genes (DEGs) in this study. The RNA-seq dataset was downloaded and processed using the Octopus-toolkit (version 2.1.0) [7]. Briefly, low-quality portions of paired-end reads were trimmed using Trimmomatic (version 0.36) [8]. The trimmed reads were mapped to the human reference genome (hg38 assembly) using STAR (version 2.5.1) [9]. The quality of the RNA-seq data was measured using the transcript integrity number (TIN) score calculated by RSeQC (version 2.6.4; tin.py) (http://rseqc.sourceforge.net/#tin-py) [10]. The abundances of whole genes (GENCODE comprehensive gene annotation, version 27; https://www.gencodegenes.org/releases/current.html) were estimated using Cuffnorm in the Cufflink application (version 2.2.1) [11]. The fragments per kilobase of transcript per million mapped reads (FPKM) values were used to compare gene's expression levels. Cuffdiff in the Cufflink program was used to identify differentially expressed genes.

### 2.2. Principal Component Analysis Plot

Principal component analysis (PCA) plots were generated using the ggfortify package in R (https://github.com/sinhrks/ggfortify) [12] with FPKM values or TIN scores. The following command was used: autoplot(prcomp(DATA), label=TRUE, label.size=3).

### 2.3. Gene Ontology Analysis and Box Plots

Gene ontology (GO) analysis was performed using Metascape (http://metascape.org/) [13]. Box plots were generated using BoxPlotR [14].

## 3. Results

### 3.1. Systematic Evaluation of RNA-Seq Data

**Figure 1:**
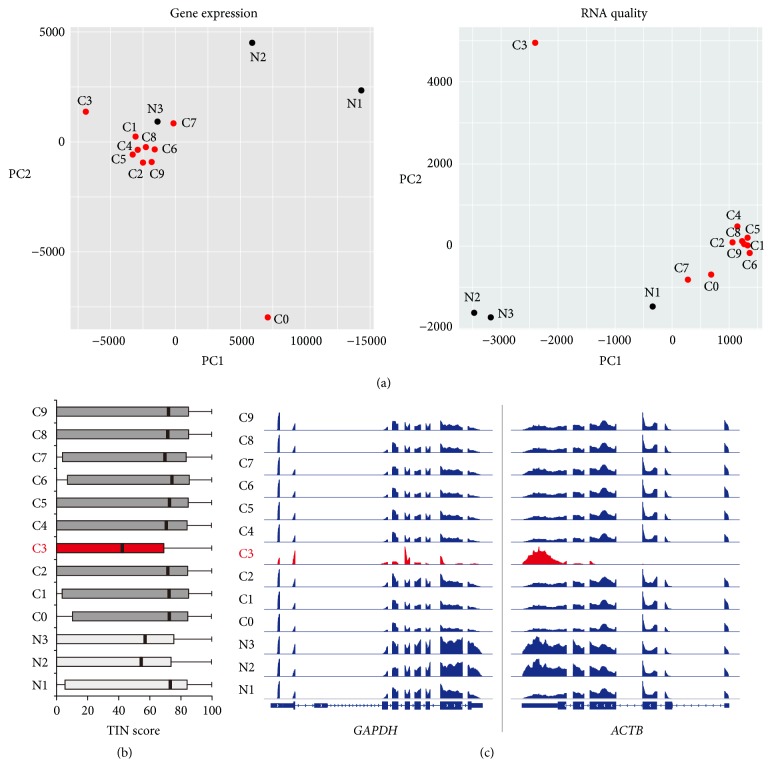
Systematic evaluation of RNA-seq data. (a) PCA plots of RNA-seq data show the characteristics of samples according to gene expression (FPKM) levels (left) and RNA quality (TIN score). Each dot indicates a sample. (b) Boxplot indicates the RNA quality of samples according to the TIN scores. A thick line (black) within the box marks the mean. (c) Genome browser snapshots of mapped read densities are shown using integrative genomics viewer (IGV). FPKM, fragments per kilobase of transcript per million mapped reads; TIN, transcript integrity number.

Although the RNA-seq technique has become a main research tool to quantify the expression levels of whole genes, researchers often do not assess the quality of RNA-seq data in detail. In fact, many studies do not determine the genuine quality (not the sequencing quality) of their RNA-seq data in depth, which could lead to the misinterpretation of results. In this study, we proposed a simple method that can assess the characteristics of RNA-seq data with mapped read files (i.e., BAM files). This approach used two estimated units, FPKM (or RPKM) and TIN score, which could be calculated easily using available applications such as Cufflinks [15] and RSeQC [10], respectively. A FPKM value [16] and TIN score [6] for a given transcript reflect expression level and RNA integrity, respectively. Therefore, the characteristics of a given RNA-seq data set can be visualized using principal component analysis (PCA) with these units. We proved the usefulness of the proposed approach by performing RNA-seq with 10 and 3 samples, respectively, obtained from invasive ductal carcinoma tissue (labeled C) and adjacent normal (labeled N) tissue of a Korean patient (GSE110114). Theoretically, the transcriptomes of cancer samples (or normal samples) in the dataset should be very similar since they were segmented from a single tissue and an expert conducted RNA-seq experiments at the same time. The characteristics of RNA-seq data were determined using the gene expression (FPKM) and RNA quality (TIN score) PCA plots (Figure 1(a)). Surprisingly, the results showed that the RNA-seq data of some of the samples were dissimilar to the other samples within the same group (cancer or normal), which indicated either differential RNA-seq quality or heterogeneous transcriptomes due to the different cell populations (Figure 1(a)). For example, the C0 sample was located far from the other cancer samples in the gene expression PCA plot, which suggested that this sample was from a spatially distinct region compared to the other cancer samples. The RNA-seq quality of the C0 sample was good, as it was positioned near the cancer sample cluster in the RNA quality PCA plot (Figure 1(a)). However, the C3 sample was located slightly outside of the cancer cluster in the gene expression PCA plot, while it was positioned far from the cancer cluster in the RNA quality PCA plot. The boxplot showing the TIN scores of samples also indicated that the C3 sample was of low quality (Figure 1(b)). Genome browser snapshots of mapped reads on two housekeeping genes,* GAPDH* and* ACTB*, also confirmed that the C3 sample was distinct from the other samples (Figure 1(c)). In the case of normal samples, the N1 sample was the highest quality, while the N2 and N3 samples were of moderate quality, as shown in the RNA quality PCA plot and boxplot (Figures 1(a) and 1(b)). Interestingly, the N3 sample was located close to the cancer cluster in the gene expression PCA plot, even though it was from adjacent normal tissue. This suggests that the majority of the transcriptomes in the N3 sample potentially came from the cancer cell population. Overall, we proposed a simple method to assess RNA-seq data in depth using the gene expression and TIN score PCA plots.

### 3.2. The Effect of Unintended Sampling for Differentially Expressed Gene Analysis

**Figure 2:**
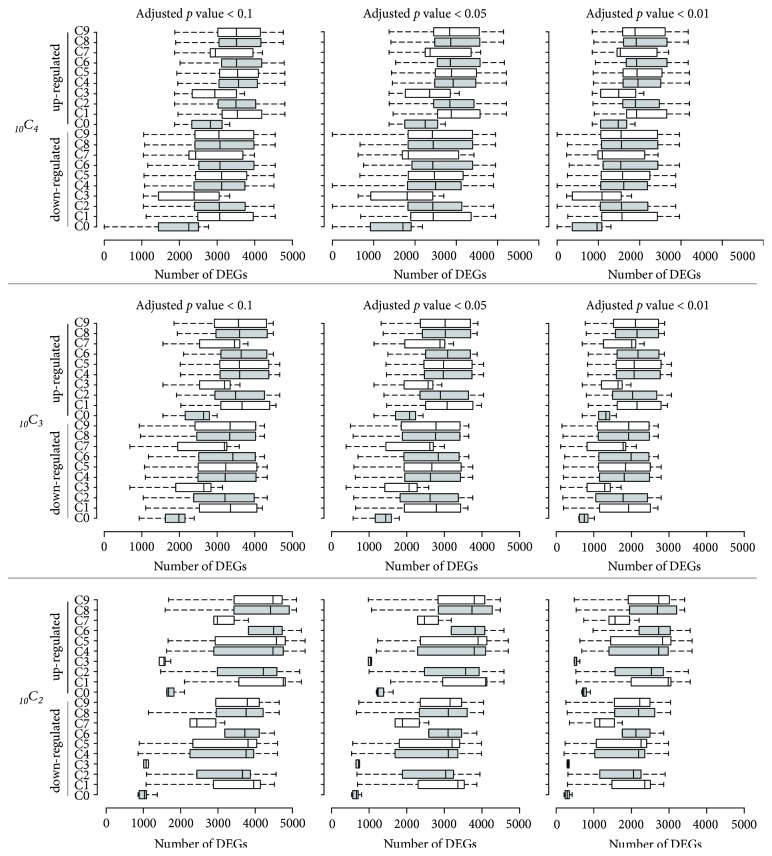
Single sample inclusion assessment for the identification of differentially expressed genes. All possible three (top panel) or two (bottom panel) cancer RNA-seq data combinations were used to identify differentially expressed genes. The x-axis shows the number of identified differentially expressed genes (DEGs) and the y-axis indicates the RNA-seq sample included in all possible combinations. The box plots show the number of DEGs identified in a given comparison setting. Centerlines show the medians and box limits indicate the 25th and 75th percentiles, as determined by R software; the whiskers extend 1.5 times the interquartile range from the 25th and 75th percentiles. The top panel has n = 36 sample points and the bottom panel has n = 9 sample points.

**Figure 3:**
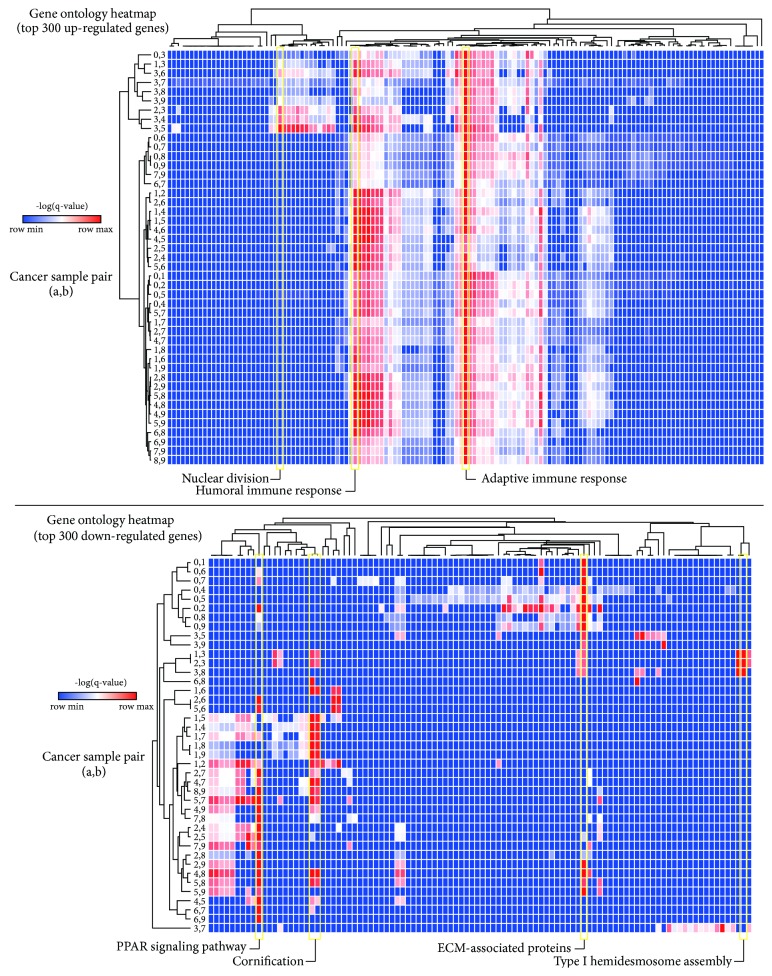
Single sample inclusion assessment of gene ontology analysis. All possible combinations of two cancer RNA-seq data were used to identify differentially expressed genes. Top 300 up- (top) or downregulated (bottom) genes were analyzed using Metascape. Significantly enriched pathways are shown in heatmaps.

One of the main purposes of conducting RNA-seq is to identify differentially expressed genes (DEGs) between given groups, which relies on statistical significance such as a false-discovery rate (FDR)-adjusted* p* value cutoff. Then, several downstream analyses such as gene ontology (GO) and network analysis are conducted to select the final gene set among identified DEGs. DEGs are generally defined as the genes that show FDR-adjusted* p* values less than a cutoff such as 0.05. We evaluated the effect of incorporating unintended samples that showed different RNA-seq qualities for DEG analysis. For this, all possible combinations of three cancer samples (or two cancer samples) were set as a cancer group and compared with three normal samples (or two normal samples: N1 and N2) using Cuffdiff with different FDR-adjusted* p* values (0.1, 0.05, and 0.01). The result showed that sampling error was critical for DEG analysis (Figure 2). For example, when a low RNA-quality sample (C3) was included to identify DEGs, the number of up- and downregulated DEGs was substantially decreased compared to other combinations. Interestingly, when the C0 sample that seemed to contain different populations of cells compared to the other cancer samples (Figure 1(a)) was included, the number of DEGs was substantially reduced. In general, the more samples that were used, the less variation was observed in the number of DEGs, even with low-quality or spatially distinct samples. The result was not substantially altered with different FDR-adjusted* p* value cutoffs (0.1, 0.05, and 0.01) (Figure 2). Next, we tested whether the sampling error could lead to a different interpretation of DEGs according to gene ontology analysis. The top 300 up- and downregulated genes were analyzed using Metascape [13] and visualized using heatmaps (Figure 3). The majority of top 300 upregulated DEGs in any given combination of two cancer RNA-seq data were associated with the adaptive immune response pathway (Figure 3). However, when a low-quality sample (C3) was incorporated as a representative sample of cancer tissue, the genes involved in nuclear division were also significantly enriched, which was not observed in the other cancer sets. Similarly, when a spatially distinct cancer sample (C0) or a low-quality sample (C3) was included as a representative of cancer tissue, the majority of top 300 downregulated genes were involved in ECM-associated proteins, whereas the other sample combinations showed that peroxisome proliferator-activated receptor (PPAR) signaling and/or cornification pathways were significantly downregulated in cancer tissues compared to adjacent normal tissues. Overall, the results showed that incorporating low-quality RNA-seq data could affect the interpretation of the RNA-seq data analysis.

### 3.3. The Quality Assessment of Publicly Available RNA-Seq Datasets

**Figure 4:**
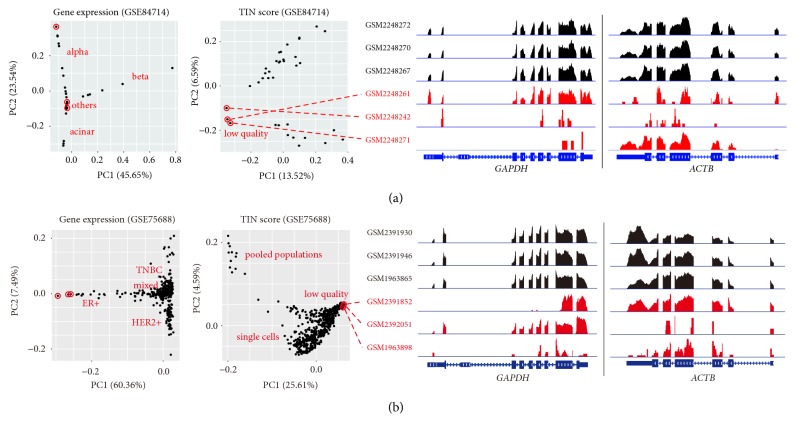
Systematic assessment of RNA-seq data. (a) PCA plots show the characteristics of islet single cell RNA-seq data (n = 36) treated with Artemether (GSE84714) using gene expression levels and RNA qualities. (b) PCA plots unveil the characteristics of breast cancer RNA-seq data (n = 563, 549 single cells and 14 bulk tumor cells) from 11 patients (GSE75688). IGV snapshots show the patterns of mapped reads on the* GAPDH* and* ACTB* genes (right panel). The proportion of variance explained is indicated in parentheses.

**Figure 5:**
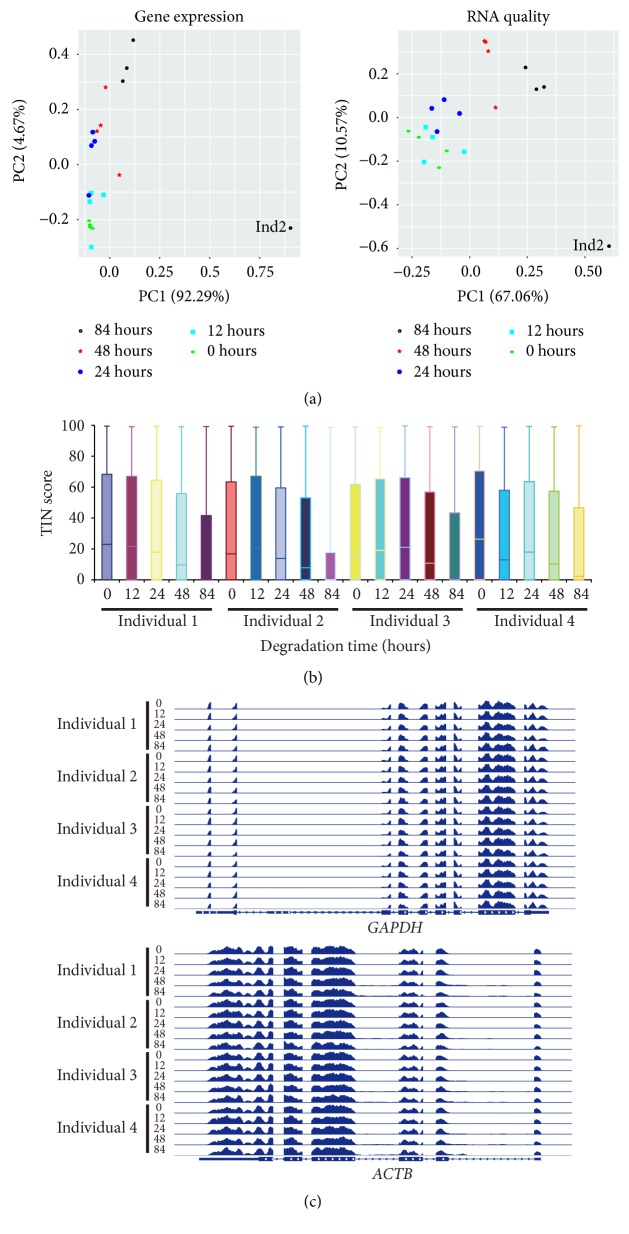
Systematic evaluation of an available RNA-seq dataset. (a) PCA plots of RNA-seq data (n = 20) show the characteristics of peripheral blood mononuclear cell (PBMC) samples that were stored unprocessed at room temperature for different time periods (0, 12, 24, 48, and 84 hours). Each dot indicates a sample. The proportion of variance explained is indicated in parentheses. (b) Boxplot indicates the RNA quality of samples according to the TIN scores. A thick line (black) within the box marks the mean. (c) Genome browser snapshots of mapped read densities are shown using integrative genomics viewer (IGV).

Since the quality of RNA-seq data can vary, we wondered whether the quality of publicly available RNA-seq datasets was acceptable according to the gene expression and TIN score PCA plots. The first RNA-seq dataset we evaluated contained 36 single-cell transcriptomes of Artemether-treated human pancreatic islets from one donor profiled by single-cell RNA-seq (scRNA-seq) technique [17]. The gene expression PCA plot mainly distinguished three cell types: alpha, beta, and acinar types (Figure 4(a)). The TIN score PCA plot classified cells into three clusters. Manual investigation of the housekeeping gene loci revealed that three single-cell transcriptomes were of low quality (Figure 4(a)). The second RNA-seq dataset we evaluated consisted of 563 RNA-seq data (549 single cells and 14 bulk tumor cells) performed with cells from 11 breast cancer patients [18]. The gene expression PCA plot showed that these single cell transcriptomes formed clusters according to their molecular subtypes: triple-negative breast cancer (TNBC), estrogen receptor positive (ER+), human epidermal growth factor receptor 2 positive (HER2+), and mixed types (Figure 4(b)). We also found some low-quality RNA-seq data, as shown in the TIN score PCA plot and genome browser snapshots of mapped reads on the housekeeping genes (Figure 4(b)). The third RNA-seq dataset contained 20 peripheral blood mononuclear cell (PBMC) RNA-seq data performed with samples that were stored at room temperature for degradation (0, 12, 24, 48, and 84 hours prior to RNA extraction) [19]. We found that one sample (PBMCs from individual 2 that were stored at room temperature for 84 hours) was of the lowest RNA quality (Figures 5(a) and 5(b)) and TIN scores were well correlated with the degree of RNA degradation as previously reported [6]. However, the patterns of mapped reads on the housekeeping genes in the sample were almost identical to other samples (Figure 5(c)), suggesting that manual investigation of the housekeeping gene loci alone is not sufficient to estimate RNA quality. In sum, the characteristics of a given RNA-seq data set can be evaluated using the combination of a gene expression PCA plot, an RNA quality PCA plot, and manual investigation of some housekeeping genes as shown in this study. For a study with a small number of samples per condition, it is recommended to use the proposed method to minimize sampling errors in RNA-seq analysis.

## 4. Discussion

All living cells contain various types of RNA; therefore, most studies focus on RNA. According to the statistics of the gene expression omnibus (GEO) database (https://www.ncbi.nlm.nih.gov/geo/summary/) at the time of writing, 67% of available data (53,279 datasets by microarray and 17,532 datasets by RNA-seq) were gene expression profiling data. Among the gene expression profiling data, the volume of RNA-seq data is rapidly increasing due to the reduced cost as well as its higher coverage and the greater resolution of the transcriptome. However, there are no simple ways to intuitively assess the characteristics of RNA-seq data. We sought to resolve this issue by proposing a simple method that could accurately characterize a given RNA-seq dataset. Without determining the characteristics of the RNA-seq data, downstream analysis such as the identification of differentially expressed genes could be significantly biased. In general, the majority of RNA-seq data registered in GEO has two or three biological replicates per condition, which means that two or three replicates represent a population for a given condition. In this scenario, if one sample is of low quality or sampling error occurred, the impact of incorporating it is significant, as shown in Figures 2 and 3. Therefore, we encourage researchers to perform the following three-step assessment: (i) draw a gene expression PCA plot to characterize the given RNA-seq data; (ii) draw a TIN score PCA plot or a TIN score boxplot to estimate the RNA quality; (iii) manually investigate some housekeeping gene loci using a genome browser application such as integrative genomics viewer (IGV) [20]. This simple approach will provide a systematic view of a given RNA-seq dataset.

Our simulation for DEG analysis highlights that the number of DEGs varies greatly when incorporating a low-quality sample or a sample from a spatially distinct region, regardless of different FDR-adjusted* p* value cutoffs. Therefore, researchers should carefully determine whether a given RNA-seq could be a representative sample of a population for a given condition with the proposed PCA plots. Our evaluation of publicly available RNA-seq data found that a few low-quality RNA-seq data had been used in original studies (Figures 4 and 5), although these low-quality RNA-seq data had not significantly affected the result in the original study because the number of biological replicates per condition was sufficiently large. However, in studies using two or three biological replicates per condition, sampling errors will be critical, leading to the misinterpretation of results. Therefore, we recommend using the proposed simple method to evaluate RNA-seq data prior to downstream analysis.

## 5. Conclusions

The proposed approach can distinguish the low-quality RNA-seq samples that should be avoided for the RNA-seq data analysis. Incorporation of low-quality RNA-seq data can lead to the misinterpretation of results as shown in the present study.

## Data Availability

RNA-sequencing data are available through the NCBI GEO database, accession code: GSE110114.
